# Dermatitis Artefacta: A Challenging Case Report

**DOI:** 10.7759/cureus.34244

**Published:** 2023-01-26

**Authors:** Lena Basfar, Ahmed Almadfaa, Bashayr A Nazer, Khalid Al Hawsawi, Shahad T Khayyat

**Affiliations:** 1 College of Medicine, Ibn Sina National College for Medical Studies, Jeddah, SAU; 2 College of Medicine, King Abdulaziz University, Jeddah, SAU; 3 Dermatology, King Abdulaziz Hospital, Makkah, SAU

**Keywords:** self-inflicted injury, rare, factitious disorder, excoriations, dermatitis artefacta

## Abstract

We report a 31-year-old female presented with a history of recurrent skin and oral lesions for 10 years. She brought a histopathology report confirming the diagnosis of pemphigus vulgaris (PV), which was found to be faked with no patient information and lacked letterhead. Skin and oral examination only reveal multiple linear upper lip erosions. We believed the patient had a preliminary diagnosis of PV, and we asked the patient to continue her medications. Based on the conflicting history and occurrence of contradictory issues, a diagnosis of dermatitis artefacta was made. The patient improved after four sessions of dialectical therapy.

## Introduction

Dermatitis artefacta (DA) is a factitious disorder in which the patient inflicts harm on himself/herself and produces skin injury. It is produced by the patient to satisfy psychological or emotional needs. It happens at the subconscious level where the patient is not consciously aware of it [1,2]. It is common in females with a ratio of 4-8:1. The age of onset is late adolescence or early adulthood [3,4]. The most common locations are any exposed body area, especially the face, upper extremities, and lower extremities. Diagnosis can be challenging. It should be considered whenever the patient shows indifference or gives an unclear or contradictory history. Patients deny having a role in creating the lesions. Establishing a good patient-doctor relationship is an important part of the management of DA [5-7]. Here, we report a case of DA who used a falsified histopathological report to achieve what she thought is right.

## Case presentation

**Figure 1:**
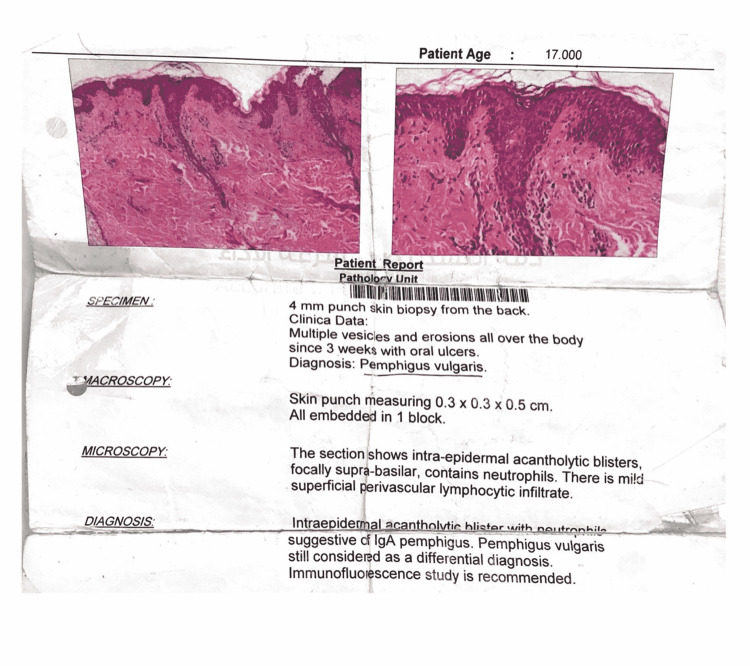
Histopathological report showing pemphigus vulgaris

**Figure 2:**
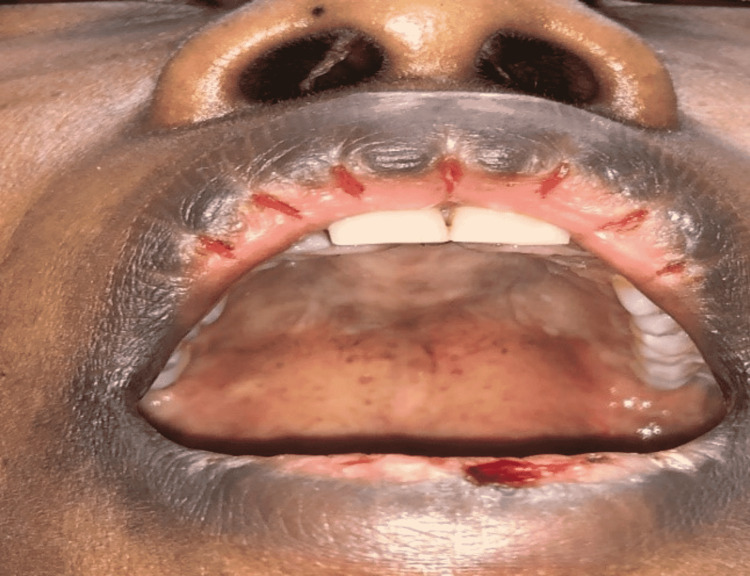
Multiple regularly arranged linear erosions over the upper lip with few crusted erosions on the lower lip

A 31-year-old female presented with recurrent skin and oral lesions for 10 years. According to the patient, a skin biopsy was taken at that time, and the diagnosis of pemphigus vulgaris (PV) was made. She brought a histopathology report confirming the diagnosis of pemphigus vulgaris (Figure 1). There were no immunofluorescence or enzyme-linked immunosorbent assay (ELISA) studies. The patient brought medications with her, including prednisolone tablets and Fucidin cream. She mentioned that these medications were prescribed for her and when she uses them, the lesions clear, but she is not regularly using them. The patient had no other significant past medical history or drug history. No history of substance abuse or suicidal thoughts or attempts. She has no history of depression. Her social history revealed that she had severe psychological and emotional upsets related to the death of her parents. Skin and oral examination didn’t reveal any lesions apart from multiple linear upper lip erosions and a few crusted erosions on her lower lip (Figure 2). On the first visit, we believed the patient, and a preliminary diagnosis of PV was made, and we asked the patient to continue her medications. During the follow-up, we noticed that the patient gives a conflicting history. In the beginning, she mentioned that she had gotten blisters in the past, but currently, she doesn’t have any blisters. Meanwhile, she sometimes mentions that she rarely gets a blister. Another contradictory issue was that the patient mentioned that she is not regular on the treatment, but when she uses the treatment, the blisters heal quickly over days to a week. This last feature is not a feature of PV. Having the previous conflicting history as well as the previous contradictory issue, we went back to the histopathology report, and we found it lacking a letterhead, and also there was no patient information. Based on the previous findings, in addition, that we have never seen any mouth or skin lesions apart from linear upper lip erosions, the presence of bel indifference in our patient where she looks happy despite mentioning that her big concern is the mouth lesions, a diagnosis of dermatitis artefacta was made. A non-judgmental, empathic patient-to-doctor relationship was built with the patient. Dialectical psychotherapy was started. The patient showed improvement after four sessions of dialectical therapy, one session every two weeks. No pharmacotherapy was needed. No more lips lesions. No more complaints of oral lesions. The patient gained insight into how she was producing the lips lesions.

## Discussion

DA is a type of factitious disorder that falls in the category of somatic symptoms and related disorders in the Diagnostic and Statistical Manual of Mental Disorders (DSM-5TM) criteria, which includes, first, deceit connected with the falsification of medical or psychological indications or symptoms, or the production of damage or sickness. Second, the individual displays themselves to others as sick, disabled, or damaged. Third, the deception is visible even when no external incentives are available. Fourth, no other mental disease, such as delusional disorder or another psychotic disorder, can explain the behavior. Our patient fulfilled all the criteria [8]. DA patients create skin lesions as a maladaptive response to psychosocial stress. They have borderline personality disorder [4,5]. DA is a long-term disorder that tends to wax and wane with the circumstances of the patient's life. The contradictory history was clear in our patient. The patient gave a conflicting history of having no blisters over the last 10 years; meanwhile, she mentions that she rarely gets blisters. We never saw any mouth or skin lesions in our patient. She also has bel indifference when she looks happy despite her big concern about the mouth lesions. The upper lip erosions that were regularly spaced were not consistent with pemphigus disease. Instead, they are typical for a factitious disorder. The most unusual presentation, in this case, was the falsification of the histopathological report. Initially, we believed in the histopathology report. However, the clues of the factitious disorder mentioned above made us go back and check the document again. The report was lacking the patient’s information and was not on letterhead paper. The history that the patient gave does not go with PV. The lesions of PV do not heal within days. Patients with DA show variable lesions according to the object that the patient used. They include abrasions, erosions, scars, ulcerations, alopecia, blisters, or bullae from burns. These lesions can resemble other inflammatory skin disorders, which makes DA difficult to diagnose [5,6]. We do not know how this patient created linear lip ulcers, but most likely, she was using a sharp tool. Patients with DA deny creating the injury deliberately; therefore, confronting these patients can complicate the underlying psychological defect [6]. DA management requires a multidisciplinary approach, including psychiatrists and psychologists. Our patient doesn’t have any manifestation of psychiatric illness, so she was not referred to a psychiatrist. Behavioral therapy proved to be helpful in the management of dermatitis artefacta [6,7]. Furthermore, the use of selective serotonin reuptake inhibitors (SSRIs) (the drugs of choice), antidepressant medication, as well as atypical antipsychotics are reported to be beneficial in some cases. However, none of these were used in our patients. We created an empathic and non-judgemental environment. We avoided confrontation with the patient. Frequent follow-ups were done to build a good patient-to-doctor relationship. During this frequent follow-up, we introduced dialectical behavioral therapy and symptomatic care for her skin lesions using petrolatum ointment. The borderline personality of our patient made her not be able to cope with the psychological trauma that was caused by the death of her parents. Dialectical behavioral therapy is a multifaceted form of cognitive-behavioral therapy that focuses on teaching patients techniques for enhancing emotion control and distress tolerance. The goal was to reduce stress and improve coping mechanisms [9]. The patient was treated by a psychologist. Dialectical behavioral therapy showed good results in our patient. After four dialectical therapy sessions, based on two-week intervals, we noticed the patient started to gain insight into the self-inflicted nature of her disorder. Our patient showed no more lip lesions and no more complaints related to her oral lesions for eight months of the follow-up. Some experts recommend that DA patients continue to see their doctor regularly for support, whether or not lesions are present. Except in mild transient cases triggered by immediate stress, the prognosis for cure is poor.

## Conclusions

Diagnosis of DA can be difficult due to obscure history and a variety of presentations. We present a case of DA mimicking pemphigus vulgaris. The case was complicated by the patient's use of a falsified histopathology report showing a diagnosis of pemphigus vulgaris; however, she didn't have other corroborating data nor clinical findings, or presentation consistent with PV. One should be vigilant about the credibility of any document the patient brings. If we were not aware of a factitious disorder, we might believe the patient, and we might continue treating the patient as a case of pemphigus. A non-confrontational and multidisciplinary approach is important for the optimum care for these patients.
